# Adding a temporal dimension to the study of Friedreich's ataxia: the effect of frataxin overexpression in a human cell model

**DOI:** 10.1242/dmm.032706

**Published:** 2018-06-25

**Authors:** Tommaso Vannocci, Roberto Notario Manzano, Ombretta Beccalli, Barbara Bettegazzi, Fabio Grohovaz, Gianfelice Cinque, Antonio de Riso, Luca Quaroni, Franca Codazzi, Annalisa Pastore

**Affiliations:** 1 Basic and Clinical Neuroscience, Maurice Wohl Institute, King's College London, 5 Cutcombe Road, London SE5 9RT, UK; 2 Division of Neuroscience, Vita-Salute San Raffaele University and IRCCS San Raffaele Scientific Institute, 20132 Milan, Italy; 3 Department of Physical Chemistry and Electrochemistry, Diamond House, Harwell Science and Innovation Campus, Didcot OX11 0DE, UK; 4 Hypha Discovery Ltd, London, UK; 5 Department of Physical Chemistry and Electrochemistry, Faculty of Chemistry, Jagiellonian University, PL-30387, Kraków, Poland; 6 Molecular Medicine Department, University of Pavia, I-27100 Pavia, Italy

**Keywords:** Frataxin, Friedreich's ataxia, Overexpression, Oxidative stress

## Abstract

The neurodegenerative disease Friedreich's ataxia is caused by lower than normal levels of frataxin, an important protein involved in iron–sulfur (Fe-S) cluster biogenesis. An important step in designing strategies to treat this disease is to understand whether increasing the frataxin levels by gene therapy would simply be beneficial or detrimental, because previous studies, mostly based on animal models, have reported conflicting results. Here, we have exploited an inducible model, which we developed using the CRISPR/Cas9 methodology, to study the effects of frataxin overexpression in human cells and monitor how the system recovers after overexpression. Using new tools, which range from high-throughput microscopy to *in cell* infrared, we prove that overexpression of the frataxin gene affects the cellular metabolism. It also leads to a significant increase of oxidative stress and labile iron pool levels. These cellular alterations are similar to those observed when the gene is partly silenced, as occurs in Friedreich's ataxia patients. Our data suggest that the levels of frataxin must be tightly regulated and fine-tuned, with any imbalance leading to oxidative stress and toxicity.

## Abstract (precis)

**Summary:** We have probed the effect of overexpression of the frataxin gene in an inducible cellular model. Our data indicate that the levels of frataxin must be tightly regulated, because any imbalance leads to oxidative stress and toxicity.

## INTRODUCTION

Friedreich's ataxia (FRDA) is a recessive, autosomal disease, with an incidence of 1 in 50,000 individuals in Caucasians (Cossee et al., 1997), characterized by progressive degeneration of large sensory neurons and cardiomyopathies (Pandolfo, 2009). It is caused by the expansion of a GAA triplet in the first intron of the *FXN* gene, which results, through epigenetic modifications upstream of the gene, in partial silencing of the gene product, frataxin, a mitochondrial protein involved in the regulation of iron-sulfur (Fe-S) cluster biogenesis (Pastore and Puccio, 2013; Sandi et al., 2013). Disease onset usually occurs at <25 years of age and shows a roughly inverse correlation with the length of the GAA expansion (Cossee et al., 1997). Typical FRDA hallmarks include mitochondrial iron accumulation, increased oxidative stress and abnormalities in Fe-S cluster biogenesis (Pandolfo and Pastore, 2009), an essential pathway involved in fold stabilization and/or providing electrons to cellular reactions.

Friedreich's ataxia is currently incurable, but several independent lines of research are being explored. Until a few years ago, the only possible palliative treatment was idebenone, an antioxidant preferentially used because of its ability to cross the mitochondrial membrane more efficiently than other less expensive antioxidants, such as vitamins C and E. Another more recent route consists of importing frataxin fused to the endocytotic TAT signal directly in the cell (Vyas et al., 2012). Although potentially attractive, this route seems to be highly inefficient, because the level of the imported protein is too low. The possibility of blocking frataxin degradation by the proteasome, interfering with the normal process of programmed cell death, was also proposed (Rufini et al., 2015). Finally, HDAC inhibitors have been considered as an effective strategy to block gene silencing. This promising direction was boosted by the development of an inhibitor that is nontoxic for the cell compared with other well-known compounds (Codazzi et al., 2016; Herman et al., 2006; Rai et al., 2010). However, although careful screening of HDAC inhibitors can be used to find the ones with lower toxicity, the intrinsic nonspecific activity of these compounds can lead to undesirable side effects on the transcription and regulation of other genes.

An important ‘if’ in all these different strategies is understanding what levels of frataxin are necessary to a healthy individual. Lack of frataxin is certainly lethal, as shown by mouse knockout models, which die at the embryonic stage (Cossee, 2000). FRDA patients have reduced but variable frataxin concentrations, and symptoms start appearing only when the frataxin level is <30% that of healthy controls (Campuzano et al., 1996). On the opposite front, experiments carried out in different cell and animal models in which frataxin was upregulated have produced conflicting results; they can be divided broadly into studies showing a harmless effect or even positive effects of frataxin overexpression (Miranda et al., 2004; Runko et al., 2008; Shoichet et al., 2002), especially in the cyto-protection from oxidative stress, and studies showing that frataxin overexpression has a detrimental effect on Fe-S biogenesis and increases oxidative stress (Llorens et al., 2007; Navarro et al., 2011; Seguin et al., 2009). Preliminary evidence also suggests that frataxin overexpression is as toxic as its partial depletion (Navarro et al., 2011). It was also reported that frataxin overexpression in a mammalian cell model is able to activate oxidative phosphorylation (OXPHOS), boost ATP production and increase mitochondrial respiration (Ristow et al., 2000). Another study based on yeast (*Schizosaccharomyces pombe*) showed that mild overexpression of the frataxin homologue Fxn1 slightly increases the oxygen consumption rate (OCR), whereas high levels of overexpression produce an 80% decrease in OCR (Wang et al., 2014). Additionally, complexes I–III in the electron transport chain (ETC) contain Fe-S clusters, the activity of which could be affected by frataxin-related disruption of Fe-S cluster biogenesis. These studies are in agreement with the model in which frataxin functions as a regulator of iron–sulfur cluster biogenesis, inhibiting in prokaryotes and activating in eukaryotes the process of conversion of cysteine into alanine through interaction with the desulfurase central to the pathway (Pastore and Puccio, 2013).

Here, we again addressed the question of which effects frataxin overexpression has on cellular metabolism using our mammalian cell line (HEK-*cFXN*), which can produce different frataxin levels by switching off the *FXN* gene (Vannocci et al., 2015). HEK-*cFXN* was genetically engineered by producing knockout of the endogenous *FXN* gene of HEK293 cells via CRISPR/Cas9 methodology and the integration in the genome of the inducible c*FXN* cassette. This system, which is the first after the exploratory study by Ristow et al. (2000) to use mammalian cells, allowed us not only to overexpress frataxin but also to introduce a temporal dimension; we monitored whether the cell recovers and how long is required for recovery from overexpression when this is removed. This information might be relevant in the development of therapeutic studies with the aim of artificially boosting the frataxin levels discontinuously. We used traditional methods and new advanced methodologies that include fluorescence and *in cell* infrared (IR). We show that frataxin overexpression is detrimental for the cell, with an appreciable increase of the reactive oxygen species (ROS) and labile iron pool (LIP) content. Partial recovery is obtained only ∼1 week after removing overexpression. Our results support a role of the protein as a regulator.

## RESULTS

### Cell model characterization

**Fig. 1.**
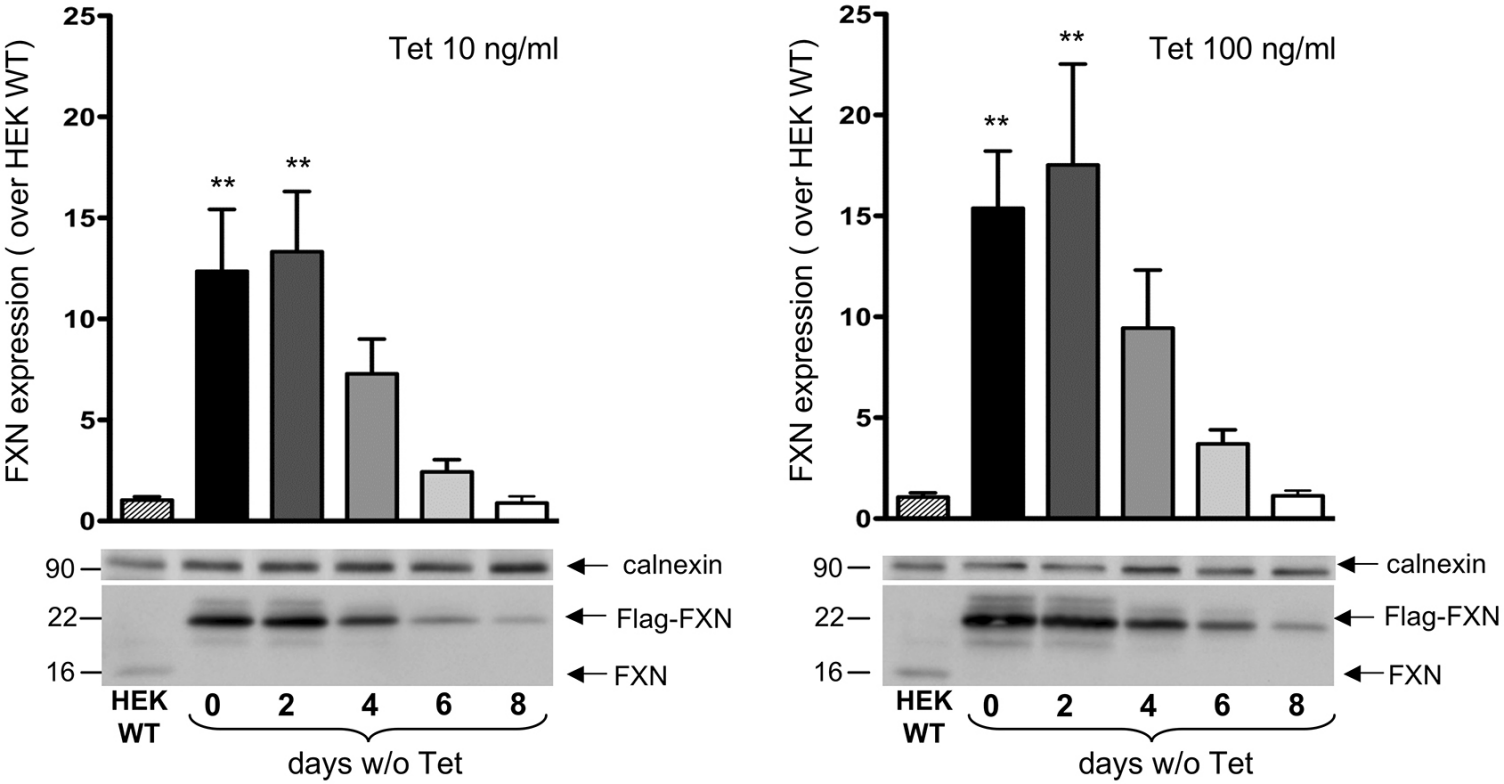
**FXN expression of the new model system.** The two graphs represent FXN expression in the presence of tetracycline (Tet) 10 and 100 ng ml^−1^, respectively [histograms corresponding to 0 days without (w/o) Tet], or after Tet removal for the indicated number of days (2, 4, 6, 8 days w/o Tet). The progressive reduction of FXN reached endogenous levels at day 8 without Tet. FXN expression was quantified (from three experiments) as folds over the endogenous FXN level in HEK293 cells (HEK WT). Data were analyzed by one-way ANOVA followed by Dunnett's post hoc test. **P≤0.01. Representative western blots of FXN and calnexin (as the loading control) are shown in the bottom panels.

The HEK-*cFXN* cellular model that we developed is characterized by a biallelic knockout of the endogenous *FXN* gene and by the presence of an exogenous, inducible cDNA *FXN* cassette (*cFXN*). The c*FXN* gene is under the control of CMV-TetO2, a tetracycline-regulated promoter, and the exogenous protein that is expressed carries at its C-terminus a 3x FLAG tag. The CMV-TetO2 promoter thus allows us to switch the frataxin gene on and off and to induce increasing levels of the protein as a function of the tetracycline concentration (Vannocci et al., 2015). When HEK-*cFXN* cells were treated with tetracycline (10 or 100 ng ml^−1^), frataxin expression significantly increased (∼13- and 17-fold increase, respectively) compared with the endogenous frataxin levels in wild-type HEK293 cells. Removal of tetracycline caused a progressive reduction of frataxin levels over an 8 day period, until physiological levels were reached at day 8 (Fig. 1).

### Frataxin overexpression and aconitase activity

**Fig. 2.**
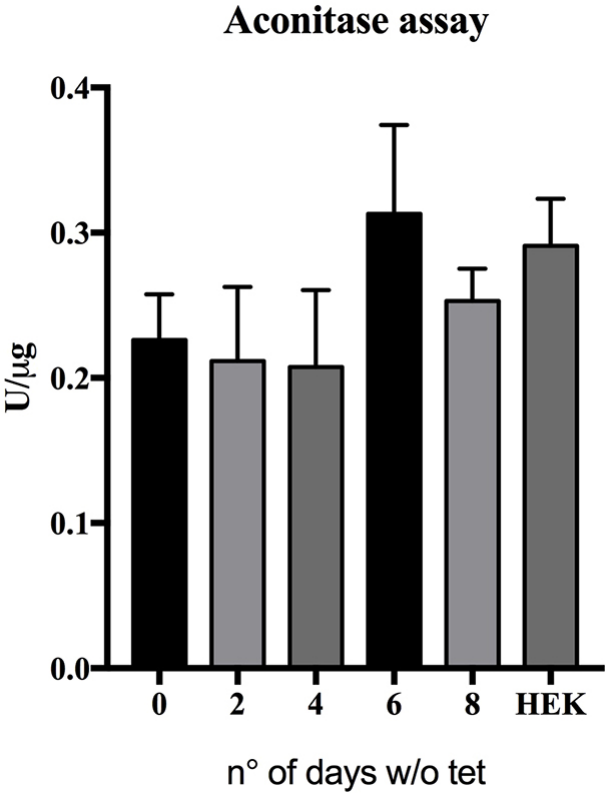
**Overexpression does not affect aconitase activity.** Results were pooled from three independent experiments done in duplicates and are expressed as means (±s.d.). Data were analyzed by ANOVA and Dunnett's post hoc test.

Although the initiation of the disease is still under debate (Pastore and Adinolfi, 2014), loss of Fe-S proteins has been widely reported as being one of the hallmarks of FRDA. Notably, reduction of aconitase activity, an important tricarboxylic acid cycle enzyme, has been consistently associated with the silencing of the *FXN* gene (Moreno-Cermeño et al., 2010; Poburski et al., 2016; Rötig et al., 1997). As a marker for the loss of Fe-S proteins, we tested the effects of overexpression of frataxin on the activity of aconitase over time. The enzyme contains a [4Fe-4S]^2+^ ion, which can be inactivated to a [3Fe-4S]^+^ cluster and catalyses the conversion of citrate to isocitrate in the citric acid cycle. *In vitro*, the reaction is moved towards an equilibrium between citrate and isocitrate, passing through the intermediate cis-aconitate in the presence of an excess of isocitrate. Aconitase activity, therefore, can be monitored by following the increase in absorbance at 240 nm associated with the formation of cis-aconitate. HEK-*cFXN* cells were initially cultured in the presence of 10 ng ml^−1^ tetracycline. The experiment was carried out by removing the antibiotic and collecting cell samples at 0, 2, 4, 6 and 8 days to monitor not only the effects of overexpression but also of its removal. In these conditions, we monitored the effect of frataxin overexpression, which returns gradually to approximately basal levels. The results showed no significant effects on the aconitase activity when the cells were cultured in the presence of tetracycline (overexpression) when compared with the activity measured in samples obtained 8 days after tetracycline removal (basal level of FXN expression; Fig. 2). Any possible effect of tetracycline on aconitase activity was excluded by performing the assay on HEK293 wild-type cells treated with either 10 or 100 ng ml^−1^ tetracycline (Fig. S1).

These results indicate that overexpression does not affect the aconitase levels compared with the control value (sample at 8 days).

### Overexpression affects mitochondrial function

**Fig. 3.**
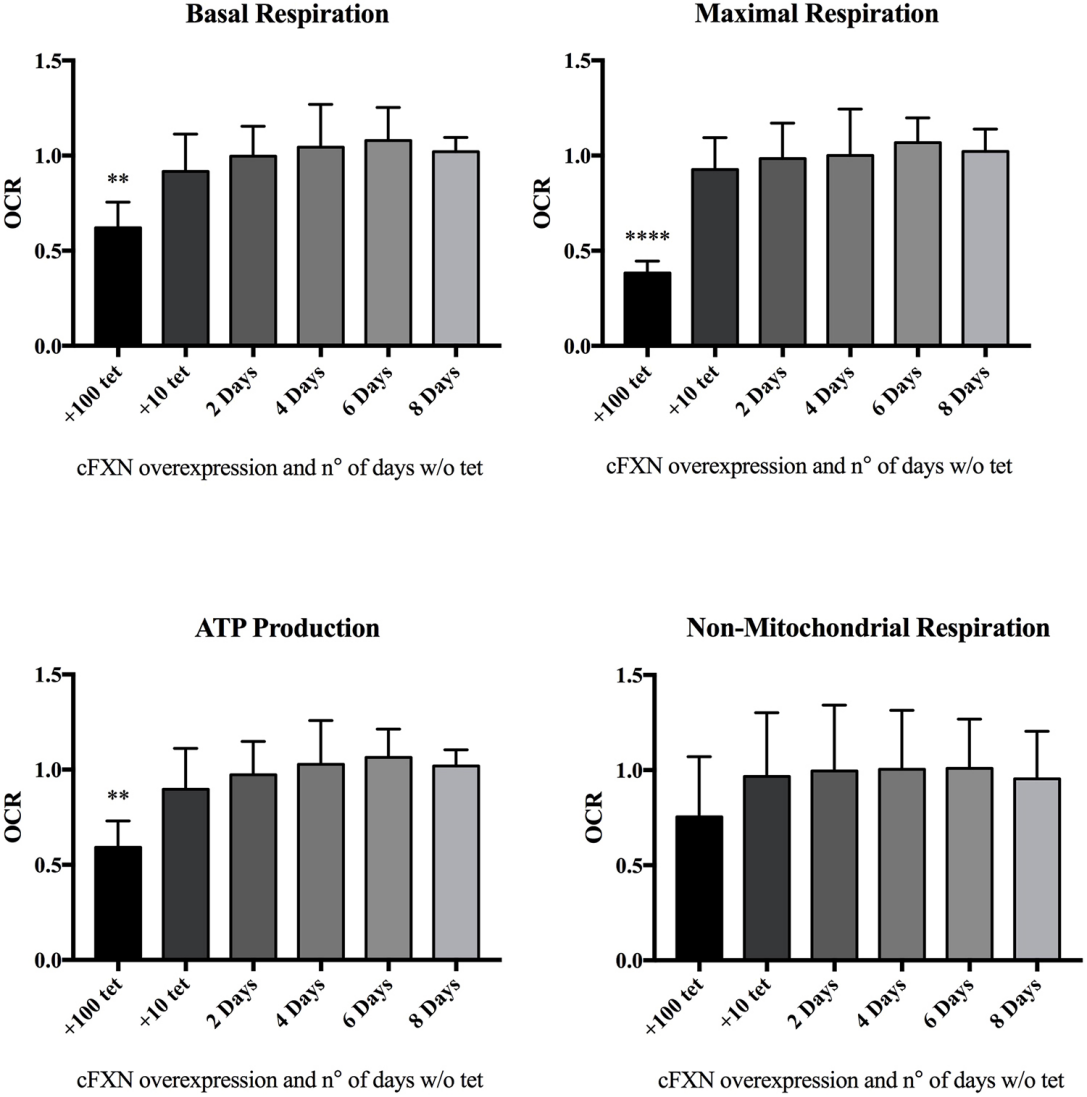
**Mitochondrial function was monitored for different levels of overexpression (10 and 100 ng ml^−1^ tetracycline) and in a time-resolved way at 0, 2, 4, 6 and 8 days on cells grown after removal of tetracycline.** The mitochondrial stress test was performed by measuring OCR variations of cells. Basal respiration, ATP production, maximal respiration and nonmitochondrial respiration were obtained by sequentially treating the samples with oligomycin, FCCP and rotenone/antimycin A compounds. Results are expressed as means (±s.d.) from five independent experiments, each with 12 technical replicates (4×10^4^ cells per sample). The recorded signals were normalized against total protein content. Data were analyzed by ANOVA and Dunnett's post hoc test. ****P≤0.0001, **P≤0.01.

We then assessed the mitochondrial function using the Seahorse XF technology (mito stress test; Agilent), an instrument which can measure OCR directly in living cells. In association with three different compounds [oligomycin, carbonyl cyanide-4-(trifluoromethoxy)phenylhydrazone (FCCP), and a mixture of rotenone and antimycin A] that are added sequentially to the medium, the measured variation in OCR can be used to assess the state of several mitochondrial functions, such as basal respiration, ATP production, maximal respiration and nonmitochondrial respiration. The mitochondrial function of HEK-*cFXN* cells was measured in cFXN overexpression conditions (10 or 100 ng ml^−1^ tetracycline) and monitored in a time-resolved way at 2, 4, 6 and 8 days on cells grown after removal of tetracycline (Fig. 3). All OCR data were normalized using the values obtained from the 8 day sample (basal level of FXN expression). In contrast to the results obtained by Ristow et al. (2000), our study did not show any beneficial effect of frataxin overexpression on ATP production or on the levels of mitochondrial respiration. We observed a significant reduction in basal respiration, ATP production and maximal respiration for cells grown in the presence of 100 ng ml^−1^ tetracycline (strong overexpression of cFXN). No differences were obtained on cells grown with 10 ng ml^−1^ tetracycline or when exploring the reduction of frataxin levels as a function of time. Any possible effect of tetracycline on mitochondrial function was excluded by performing the Seahorse assay on HEK293 wild-type cells treated with either 10 or 100 ng ml^−1^ tetracycline (Fig. S2). These results indicate that overexpression of frataxin does not confer any beneficial advantage to mitochondrial function.

### Frataxin overexpression strongly affects oxidative stress and iron content

**Fig. 4.**
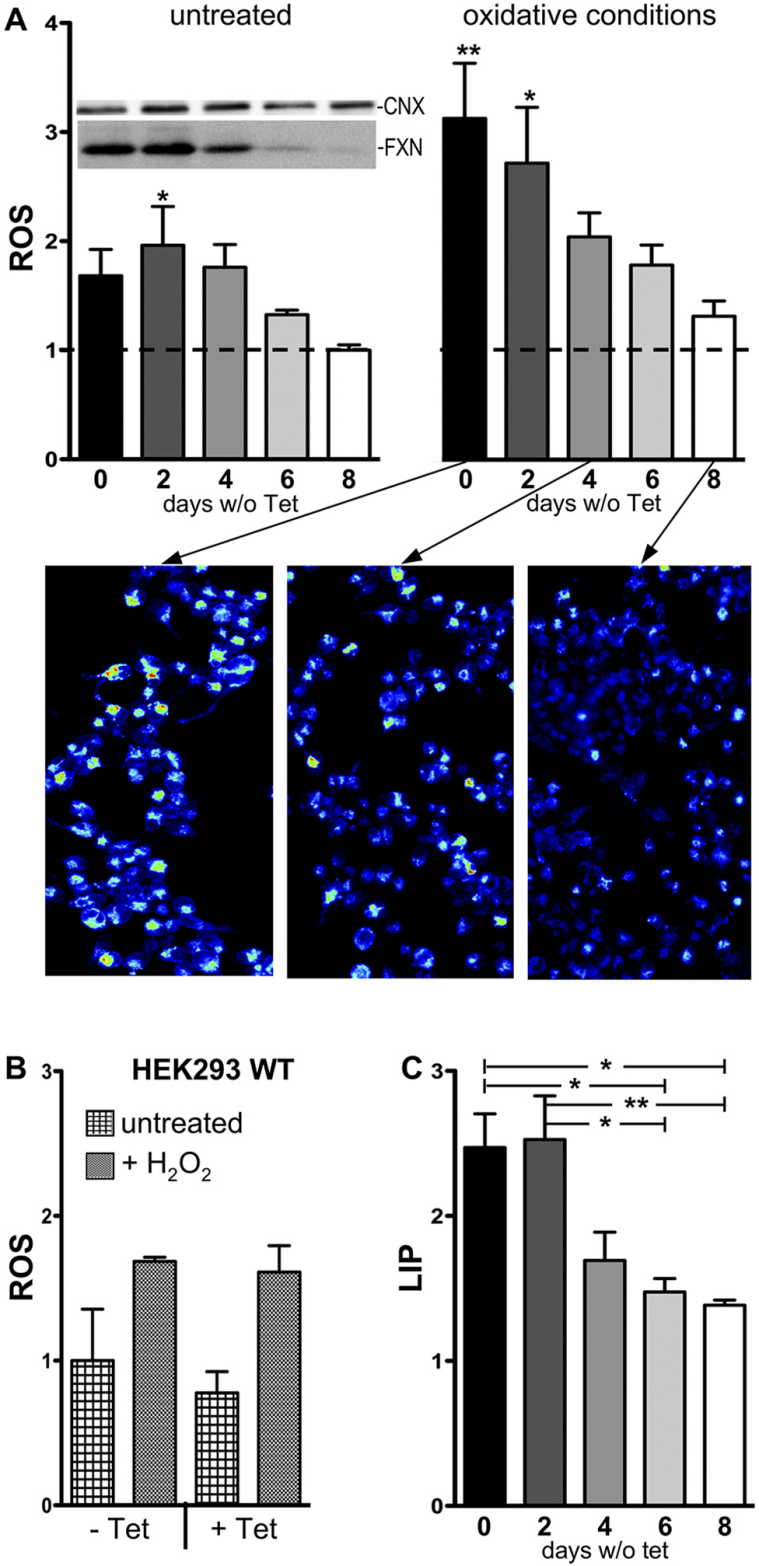
**HEK-*cFXN* cells show a correlation among ROS levels, LIP and frataxin expression.** (A) The two graphs represent ROS levels analyzed concomitantly on HEK-*cFXN* maintained under the indicated conditions and normalized for the lowest value [in untreated cells, kept 8 days without tetracycline (Tet; dashed line)]. The inset in the left panel represents the expression of frataxin at the corresponding time points of Tet deprivation. Oxidative conditions were obtained by overnight treatment with ferric iron (50 μM ferric ammonium citrate) combined with acute exposure to hydrogen peroxide (15 min, 300 μM). The bars correspond to the mean (±s.e.m.), from three independent experiments (each condition in duplicate; >5000 cells per condition), of CellROX fluorescence signals. Representative fluorescence images from the time points indicated by the arrows are shown in the lower panel. Statistical significance was calculated using one-way ANOVA followed by Dunnett's post hoc test; *P<0.05, **P<0.01. (B) ROS levels, analyzed in wild-type HEK293 (HEK293 WT) were not affected by Tet (10 ng ml^−1^) treatment, even after hydrogen peroxide (15 min, 300 μM). (C) LIP was evaluated as the fold increase of calcein fluorescence after administration of 100 μM SIH, compared with before. Statistical significance was calculated using one-way ANOVA followed by Bonferroni post hoc test; *P<0.05, **P<0.01.

The effects of frataxin overexpression on ROS generation and LIP levels were analyzed in the culture conditions described above, i.e. cells maintained for the indicated number of days in the absence of 10 ng ml^−1^ tetracycline. Frataxin expression was evaluated by western blots in parallel with ROS and LIP at the same time points. Measurements of ROS were performed not only in untreated cells, but also under oxidative conditions to mimic the oxidative status chronically present in patients. For this purpose, the cells were subjected to a protocol of mild chronic iron overload (maintained overnight in the presence of 50 μM of ferric iron) in combination with acute administration of hydrogen peroxide (300 μM, 15 min). ROS generation, studied either in the absence or in the presence of hydrogen peroxide, promotes the oxidation of CellROX dye, the fluorescence of which is proportional to the oxidation levels. All data were normalized for fluorescence signals obtained at day 8, when both frataxin expression and ROS levels are the lowest, to highlight the effect of *cFXN* overexpression on ROS production. This analysis shows a progressive and significant decrease of ROS levels (Fig. 4A) that is directly correlated with frataxin expression (see western blot inset in the upper panel). Frataxin overexpression makes the cells particularly susceptible to oxidative conditions; of note, the ROS levels were also higher in untreated cells, suggesting that frataxin overexpression promotes oxidative stress per se. ROS production was not affected by simple tetracycline treatment, because wild-type HEK293 cells grown in the presence or in the absence of the antibiotic (10 ng ml^−1^) showed the same ROS levels, even after acute treatment with hydrogen peroxide (300 μM, 15 min; Fig. 4B).

Alterations of frataxin expression might affect iron homeostasis, not only at the mitochondrial level but also at the cytosolic level, with potential consequences on oxidative stress. Therefore, the effects of frataxin overexpression on LIP content were analyzed, by the calcein fluorescent dye, at the same time points as ROS measurements. Experiments were performed in the same conditions of mild iron overload, because the sensitivity of the assay makes the measurements of basal LIP unreliable (not shown). Interestingly, in these conditions, LIP was significantly higher in cells overexpressing frataxin (0 and 2 days without tetracycline), indicating their reduced competence in the control of cellular iron handling (Fig. 4C). Comparable results, in terms of ROS production and LIP levels, were obtained when the cells were maintained in 100 ng ml^−1^ of tetracycline (data not shown). We can therefore conclude that frataxin overexpression promotes oxidative stress and alteration of cellular iron homeostasis.

### *In cell* IR spectroscopy indicates strong metabolic changes

**Fig. 5.**
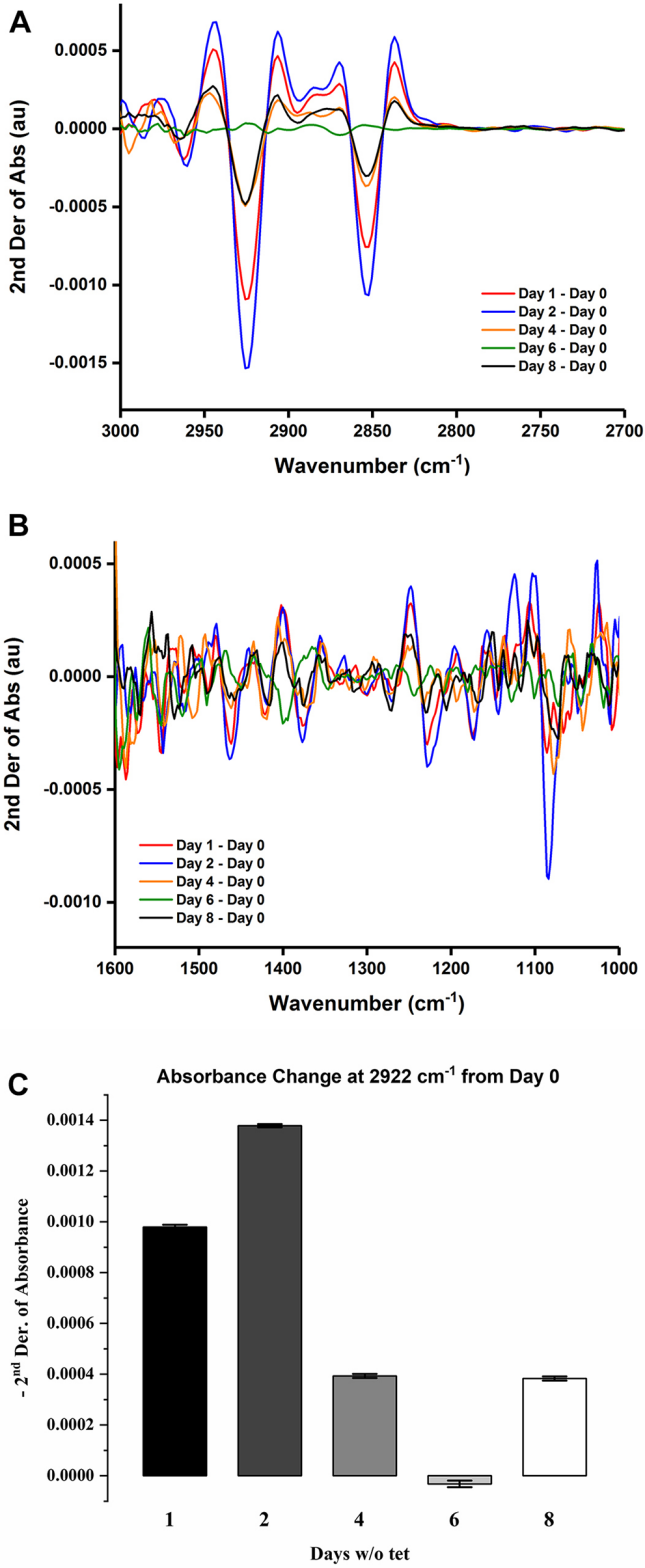
***In cell* IR measurements.** (A) Difference spectra of HEK293-*cFXN* cells after removal of tetracycline (tet). (B) Second derivative of absorbance from spectra normalized to the 1545 cm^−1^ band. (C) Time evolution of absorption variation at 2922 cm^−1^ after removal of tetracycline. Bars represent the s.d.

We measured the *in cell* IR absorption spectra at the same time points. The average IR spectra were normalized to the band at 1545 cm^−1^. In a fixed cell, absorbance in this position is commonly attributed to amide bonds, for example peptide bonds and some amino acid side-chains, but also acetylated polysaccharides (Arrondo and Goñi, 1999; Barth, 2000). In a live sample, absorption bands from some metabolites also provide a contribution to the absorption at this frequency (Quaroni and Zlateva, 2014). It is commonly accepted, although not always verified, that the contribution from proteins is dominant. The strongest variations over time were observed for a doublet of sharp bands at 2925 and 2853 cm^−1^ (Fig. 5A), which can be assigned to long alkyl chains, such as the acyl chains of phospholipids and triglycerides. Variations of the phospholipid content are also supported by variations at 1743 and 1718 cm^−1^ (not shown), which correspond to the bands of carbonyl groups of esters and protonated carboxylic acids. A complex series of changes was also observed in the 1000–1600 cm^−1^ range (Fig. 5B).

Given that band assignment in this region is complex in live cell samples because of the presence of multiple and overlapping bands, we carried out a correlated cellular microscopy (CSM) analysis. CSM relies on two-dimensional correlation spectroscopy (2DCOS) (Quaroni and Zlateva, 2014; Quaroni et al., 2011) to assign complex band patterns in cellular spectra that evolve over time, based on the correlation of their changes. The bands that evolve in synchrony are clustered together (Fig. S3). CSM allowed us to identify two main groups of bands that change as a function of time (Fig. 5B). One set corresponds to the absorption bands of phospholipids (1084, 1174, 1225, 1375 and 1460 cm^−1^), together with other molecules that are likely to be carboxylate- or alkyl ammonium-containing molecules, including metabolites and amino acid side-chains (1542, 1575 and 1585 cm^−1^). This can be interpreted as a change in the lipid to protein ratio. Interestingly, an altered lipid to protein ratio was already reported in both *Drosophila* and mouse FRDA models (Chen et al., 2016a,b; Martelli et al., 2012; Navarro et al., 2010). Changes in the opposite direction of another set of bands (1028, 1098, 1105, 1125, 1155, 1248, 1355, 1402, 1435 and 1480 cm^−1^) are assigned to the combined contribution of several molecules, such as metabolites (Quaroni and Zlateva, 2014; Quaroni et al., 2016). The changes indicate major variations in cellular metabolism and suggest an overall alteration of the protein to phospholipid ratio.

The effect occurs during the first 2 days after tetracycline removal. It then returns to the initial value (time 0) around day 6 and increases thereafter. The changes cannot be ascribed only to differences in frataxin levels; comparison of the average spectra of cell cultures grown with different levels of frataxin overexpression (10 or 100 ng ml^−1^ of tetracycline) show only minimal differences (Fig. S4), as expected from the relative contribution of frataxin to the total proteome. We can interpret these results as an initial change in overall protein expression, which is then compensated by activation of different pathways over the next 2 days, leading to renewed rates of protein synthesis. These experiments thus reveal as yet unidentified aspects of imbalances of the frataxin levels.

## DISCUSSION

Here, we have studied the effects of different frataxin levels in a cellular model. This knowledge is needed to understand how to fine-tune the protein levels during the development of therapies that could be used for the treatment of FRDA. We probed different cellular functions using different advanced techniques and monitoring the temporal progression towards physiological levels of frataxin. We found that strong overexpression of frataxin does not confer any beneficial advantage on mitochondrial function. On the contrary, a strong overexpression (100 ng ml^−1^ tetracycline) leads to a significant reduction in ATP production and basal and maximal respiration. The negative effects of cFXN overexpression are even more striking on oxidative stress and LIP. Iron dysregulation was observed solely in conditions of highly elevated frataxin overexpression, in which the mild alterations in mitochondrial function could hardly be the cause. LIP elevation can, at least in part, account for the generation of oxidative stress, although ROS production also remains elevated when LIP returns to the basal level, suggesting that other mechanisms might be involved, as proposed below. Finally, we have also proved the feasibility of studies based on new and complementary techniques, such as *in cell* IR.

It is appropriate to compare our results with those obtained using other animal and/or cellular models. The first similar study dates back to 2000, when Ristow et al. (2000) showed that frataxin overexpression in mammalian cells causes a calcium-induced upregulation of tricarboxylic acid cycle flux and respiration, which results in an increased mitochondrial membrane potential and an increase of cellular ATP levels. The authors concluded that frataxin plays an important role in mitochondrial energy conversion and oxidative phosphorylation. Seguin et al. (2009) looked at different aspects in a *Saccharomyces* *cerevisiae* model. They showed that mitochondrial iron is more available to ferrochelatase in frataxin-overexpressing yeast, resulting in higher levels of haem synthesis *in vitro*. They also showed that overexpression results in a shift from frataxin trimers to oligomers of higher molecular mass in the mitochondrial matrix and that, surprisingly, the overexpressed cells are more resistant to oxidizing agents than wild-type cells (Seguin et al., 2009). Runko et al. (2008) showed that overexpression of frataxin in *Drosophila* increases antioxidant capability and resistance to oxidative stress insults and results in longevity. These results suggest that *Drosophila* frataxin might function as a factor that protects mitochondria from oxidative stress and cellular damage.

Navarro et al. (2011) obtained transgenic *Drosophila* flies that overexpressed human or fly frataxin using the UAS-GAL4 system. The authors observed deleterious effects at the biochemical, histological and behavioural levels for both model systems and increased levels of oxidative stress. They also proved that frataxin overexpression reduces *Drosophila* viability and impairs the normal embryonic development of muscle and the peripheral nervous system, with reduced levels of aconitase activity. Frataxin overexpression in the nervous system reduces lifespan, impairs locomotor ability and causes brain degeneration (Navarro et al., 2011). Most recently, Wang et al. (2014) showed that overexpression of the frataxin homologue fxn1 under a thiamine repressible promoter in an *S. pombe* model results in a phenotype strongly dependent on the amount of Fxn1 overexpression. They showed that high Fxn1 overexpression inhibits *S. pombe* growth and impairs mitochondrial membrane integrity and cellular respiration. It also leads to Fxn1 aggregation and cellular iron accumulation. Defence mechanisms against oxidative stress and mitochondrial Fe-S cluster-containing enzyme activities proved to be upregulated upon overexpression. The authors concluded that dysregulated Fe-S cluster biogenesis is a primary effect that is shared by both frataxin overexpression and deficiency (Wang et al., 2014).

The discrepancies between these results could be rationalized by considering that the animal models used in these studies are very different, as are the methodologies and the assays. Overall, our data represent the only example, after the work of Ristow et al. (2000) in the very early days of investigation of frataxin, from mammalian cells. Nevertheless, contrary to this previous study, we do not find that frataxin overexpression results in a significant increase in ATP. We show that mitochondrial function is differently affected by increasing levels of *FXN* overexpression; therefore, the discrepancy could be attributable to differences in the levels of *FXN* overexpression achieved in the two models. It is also possible that in our cell system the elevation of mitochondrial calcium content observed by Ristow et al. (2000) does not activate OXPHOS and ATP production but, instead, might synergistically contribute to increase the oxidative stress level. In fact, it has been reported that silencing of mitochondrial calcium uniporter (MCU) and consequent reduction of mitochondrial Ca^2+^ entry plays a protective role in HeLa cells and in cerebellar granule neurons by decreasing the susceptibility to oxidative stress conditions (Liao et al., 2015).

Our results are closer to those of Navarro et al. (2011) and strongly suggest that both high and low levels of frataxin induce oxidative stress. We also find no amelioration of the phenotype as a consequence of overexpression, as deduced by Runko et al. (2008). On the contrary, the increase of LIP not only promotes ROS production, but might also alter other intracellular pathways that depend on cellular iron content, as previously suggested (Chen et al., 2016b), thus contributing to cellular toxicity. Neuronal cells, which are affected in FRDA, are indeed more susceptible to oxidative insults and other stressful conditions (Pelizzoni et al., 2011).

Our results might be important for the design of therapeutic strategies. Gene therapy is one of the most promising approaches for curing this autosomal disease, which is caused by loss of function as a consequence of partial silencing of the *FXN* gene. However, the traditional method of randomly integrating an exogenous, therapeutic *FXN* gene in the genome to restore protein levels could potentially backfire because of the risk of insertional oncogenesis (Hacein-Bey-Abina et al., 2008, 2010). In this regard, the development of non-integrating viral vectors, such as adeno-associated viruses (AAVs), has greatly improved the safety of gene therapy. This approach has been used successfully with remarkable results in two conditional mouse models that mimic the cardiomyopathy and neuronal dysfunction of FRDA (Gérard et al., 2014; Perdomini et al., 2014). After AAV delivery, frataxin levels were increased in the relevant tissues, and both mouse models showed increased life expectancy and a great reduction in FRDA-associated phenotypes (Gérard et al., 2014; Perdomini et al., 2014). Both viral approaches, however, take advantage of a human exogenous *FXN* gene under the control of strong promoters that induce overexpression of the therapeutic genes. Although the mouse models showed great improvements, the lack of a tight control on the levels of expression could generate the effects detailed in the present study, with unknown long-term consequences for patients treated this way. Recent studies have taken advantage of the new CRISPR gene editing approach to produce the desired gene correction (Ouellet et al., 2017). As an alternative, gene correction of the endogenous *FXN* gene by reduction of the GAA expansion seems to be preferable. This strategy has the advantage that frataxin levels would be restored to physiological levels. It is, however, essential for these studies to determine the effects of different levels of frataxin.

Finally, our conclusions strongly support the view of frataxin as a regulator. Frataxin seems to function as the green light that senses the effector iron concentration and allows cluster formation only when sufficient concentrations of acceptors are available (Prischi et al., 2010). So far, this model has been supported fully by independent lines of evidence. We have also recently demonstrated that another protein of the *isc* operon, YfhJ (also called IscX), which is present almost exclusively in prokaryotes, modulates the effect of frataxin in bacteria and blocks its inhibitory power as a function of iron concentrations (Pastore et al., 2006). In eukaryotes, the desulfurase is inactivated, and frataxin acts as an activator of the same process according to an adaptation program that is often observed between prokaryotes and eukaryotes. This evidence, together with what we have reported in the present study, strongly supports the view that frataxin is a regulator for which levels must be tightly controlled and fine-tuned to the concentrations of iron and cluster acceptors at any time point, with any imbalance leading to oxidative stress and toxicity.

## MATERIALS AND METHODS

### Experimental outline

All measurements were carried out using the same experimental set-up. Expression or overexpression of frataxin was obtained by culturing HEK-*cFXN* cells in the presence of 10 or 100 ng ml^−1^ tetracycline. Experiments designed to monitor the effects of the reduction of frataxin levels over time were performed by culturing cells in the presence of 10 ng ml^−1^ tetracycline, followed by removal of the antibiotic from the medium. Measurements were carried out in each experiment at specific time points (2, 4, 6 and 8 days after turning off *cFXN* expression).

### Cell culture

Cell culture media and reagents, if not otherwise stated, were from Thermo Fisher Scientific (Waltham, MA, USA). Wild-type HEK293 and HEK-*cFXN* cells were cultured in Dulbecco's modified Eagle's medium, supplemented with 10% tetracycline-free fetal bovine serum (FBS; Clontech, Takara), 10 mM sodium pyruvate, 2 mM l-glutamine, 50 U ml^−1^ penicillin and 50 µg ml^−1^ streptomycin and 2% (v/v) non-essential amino acids (NEAAs), and kept at 37°C in humidified air enriched with 5% CO_2_. The culture medium for HEK-*cFXN* cells was supplemented with 100 µg ml^−1^ hygromycin B, 15 µg ml^−1^ blasticidin, 0.4 µg ml^−1^ puromycin and varying concentrations of tetracycline (Sigma-Aldrich), depending on the desired level of frataxin induction (0, 10 or 100 ng ml^−1^).

### Western blots

Cell samples were collected, pelleted by centrifugation and resuspended in 0.01 M sodium EDTA, 0.2% SDS and 2% NP40 in PBS, with protease inhibitors (chymostatin, leupeptin, antipain and pepstatin 1:1000). Samples were lysed for 30 min at 4°C with shaking, centrifuged for 10 min at 13,000 **g** to remove the pellet, and the protein concentration in the supernatant was measured using Pierce BCA assay (Thermo Fisher Scientific). Proteins (15 or 30 µg) were denatured for 5 min at 95°C in Laemmli sample buffer and subjected to SDS-PAGE using 12.5% polyacrylamide gels (Life Technologies). Samples were then transferred to a nitrocellulose membrane (0.45 μm pore size; GE Healthcare Lifescience). After 1 h blocking with TBS, 0.1% Tween-20 (Promega) and 1% milk, the membranes were incubated overnight at 4°C with primary antibodies. Membranes were then washed with TBS and 0.1% Tween and incubated at room temperature for 1 h with either goat anti-mouse or anti-rabbit secondary antibody (Bio-Rad), depending on the primary antibody. The reaction was carried out with secondary horseradish peroxidase conjugates and enhanced chemiluminescence detection (Pierce Supersignal). Immunoblot bands were analyzed by Chemidoc Touch Imaging System (Bio-Rad). Calnexin was used to normalize values. During the project, we used the following primary antibodies: mouse anti-FLAG M2 antibody (1:10,000 dilution; F1804, Sigma-Aldrich), rabbit anti-GAPDH (1:5000 dilution; 2118S, Cell Signaling Technologies), mouse anti-Tom20 (1:5000 dilution; 612278, BD Biosciences), anti-frataxin (1:3000 dilution; kindly gifted by Dr F. Taroni, from Carlo Besta Neurological Institute, Milan) and anti-calnexin (1:2500 dilution; BD Biosciences).

### Aconitase assay

Whole-cell aconitase enzymatic activities were measured spectrophotometrically using an aconitase enzyme activity kit (Abcam; ab109712) following the manufacturer's guidelines. Briefly, 50 μg of total protein from whole-cell lysates was resuspended in aconitase preservation buffer and loaded on a 96-well microplate. Equal amounts of the substrate isocitrate and manganese were added to all wells, and the absorbance at 240 nm was recorded every 20 s for 30 min. The catalytic activity was measure by the rate of formation of cis-aconitate as detected by the increase in absorbance between 10 and 20 min (two time points between which the rate is increasing linearly for all samples). The change in absorbance per minute was converted to units per microgram (specific activity), i.e. the activity of the enzyme per microgram of total protein expressed as millimoles per minute per microgram.

### Seahorse mitochondrial stress test

HEK293 cells and HEK-*cFXN* cells were plated into Seahorse 96-well XF cell culture microplates (cat. no. 101085-004) at a density of 4×10^4^ cells per well using medium supplemented with the relevant antibiotics and different concentrations of tetracycline (10 and 100 ng ml^−1^). Cells were left overnight at 37°C in humidified air supplemented with 5% CO_2_. The next day, the medium was changed to XF Base Medium (cat no. 102353-100) supplemented with 1 mM pyruvate, 2 mM Glutamax and 10 mM glucose (pH of the medium was adjusted to 7.4 using 1 M NaOH). Cells were then incubated for 1 h at 37°C in a CO_2_-free incubator. The XF Cell Mito Stress Test (cat no. 103015-100) was carried out by loading the XF 96 Extracellular Flux assay sensor cartridge with oligomycin (final well concentration 1 µM), FCCP (final well concentration 0.25 µM) and rotenone/antimycin A (final well concentration 0.5 µM). Readings were normalized against the total protein content of each sample, and each reading was reported as the ratio to the measurement at day 8 (without tetracycline). Statistical significance was tested using one-way ANOVA and Dunnett's post hoc test (**P<0.01; ****P<0.0001).

### Fluorescence microscopy measurements

For ROS and LIP measurements, tetracycline (10 or 100 ng ml^−1^) was removed from the medium at the indicated number of days, and the cells were plated on 96-well plates 48 h before fluorescence microscopy measurements. Dye loading and fluorescence analyses were performed in Krebs Ringer Hepes buffer (KRH; mM: 5 KCl, 125 NaCl, 2 CaCl_2_, 1.2 MgSO_4_, 1.2 KH_2_PO_4_, 6 glucose and 20 Hepes, pH 7.4) at 37°C. Cells were loaded with CellROX Orange Reagent (5 μM, 30 min; Thermo Fisher Scientific) and with calcein AM (0.25 μM, 10 min; Thermo Fisher Scientific) for ROS and LIP measurements, respectively. In particular, for LIP analysis, the fluorescence was measured before and 10 min after incubation with 100 μM salicylaldehyde isonicotinoyl hydrazone (SIH), a cell-permeant iron chelator, according to Kakhlon and Cabantchik (2002). Nuclear staining, necessary for image acquisition and analysis, was performed with Hoechst 33342 for 10 min at a final concentration of 10 µg ml^−1^. Images were acquired with an automated epifluorescent inverted microscope (Array Scan XTI platform; Thermo Fisher Scientific). Statistical significance was tested using one-way ANOVA followed by either Dunnett's post hoc test for ROS or Bonferroni's post hoc test for LIP analyses, respectively; *P<0.05; **P<0.01.

### *In cell* IR

Cells were grown on 25 mm, 1-mm-thick, CaF_2_ optical windows (Crystran, UK) to give extended adherent layers. The sample holder for Fourier-transform infrared spectroscopy imaging on live cells is based on a sandwich design, with two optical windows separated by a spacer. The window with adherent cells is placed in the holder before being moved to the IR microscope for the measurement. The sample temperature is set at 37°C by connection to a circulating water bath (Lauda, AT).

IR measurements were performed on a Bruker Vertex 80v interferometer (Bruker Optics, D) connected to a Bruker Hyperion 3000 IR microscope with a 64×64 pixel FPA MCT detector. A Ge-multilayer KBr beamsplitter and the internal globar lamp were used to measure in the mid-IR spectral region. An extended monolayer of cells was selected for the measurement using the visible optics of the microscope. The background measurement was performed on a region of the sample where only medium was present. Two hundred and fifty-six scans were used for the background and 128 for the sample. Scans were run in double-sided forward–backward acquisition mode at a scan rate of 2.5 kHz and 4 cm^−1^ resolution. Single channel spectra were obtained by performing a Fourier transform of the interferogram after apodization with a Blackman–Harris 3-term function, using a zero-filling factor of two and a Mertz phase correction. Data were plotted as absorbance spectra. All the pixels of each spectral hypercube were averaged to obtain the spectrum of a cell layer. Samples were run in triplicate except for day 6, which was run in duplicate.

## Supplementary Material

Supplementary information

## References

[DMM032706C1] Arrondo J. L. R. and Goñi F. M. (1999). Structure and dynamics of membrane proteins as studied by infrared spectroscopy. *Prog. Biophys. Mol. Biol.* 72, 367-405. 10.1016/S0079-6107(99)00007-3 10605294

[DMM032706C2] Barth A. (2000). The infrared absorption of amino acid side chains. *Prog. Biophys. Mol. Biol.* 74, 141-173. 10.1016/S0079-6107(00)00021-3 11226511

[DMM032706C3] Campuzano V., Montermini L., Molto M. D., Pianese L., Cossee M., Cavalcanti F., Monros E., Rodius F., Duclos F., Monticelli A. et al. (1996). Friedreich's ataxia: autosomal recessive disease caused by an intronic GAA triplet repeat expansion. *Science* 271, 1423-1427. 10.1126/science.271.5254.1423 8596916

[DMM032706C4] Chen K., Ho T. S., Lin G., Tan K. L., Rasband M. N. and Bellen H. J. (2016a). Loss of Frataxin activates the iron/sphingolipid/PDK1/Mef2 pathway in mammals. *Elife* 5, e20732 10.7554/eLife.20732 27901468 PMC5130293

[DMM032706C5] Chen K., Lin G., Haelterman N. A., Ho T. S., Li T., Li Z., Duraine L., Graham B. H., Jaiswal M., Yamamoto S. et al. (2016b). Loss of Frataxin induces iron toxicity, sphingolipid synthesis, and Pdk1/Mef2 activation, leading to neurodegeneration. *Elife* 5, e16043 10.7554/eLife.16043 27343351 PMC4956409

[DMM032706C6] Codazzi F., Hu A., Rai M., Donatello S., Salerno Scarzella F., Mangiameli E., Pelizzoni I., Grohovaz F. and Pandolfo M. (2016). Friedreich ataxia-induced pluripotent stem cell-derived neurons show a cellular phenotype that is corrected by a benzamide HDAC inhibitor. *Hum. Mol. Genet.* 25, 4847-4855. 10.1093/hmg/ddw308 28175303

[DMM032706C7] Cossee M. (2000). Inactivation of the Friedreich ataxia mouse gene leads to early embryonic lethality without iron accumulation. *Hum. Mol. Genet.* 9, 1219-1226. 10.1093/hmg/9.8.1219 10767347

[DMM032706C8] Cossee M., Schmitt M., Campuzano V., Reutenauer L., Moutou C., Mandel J.-L. and Koenig M. (1997). Evolution of the Friedreich's ataxia trinucleotide repeat expansion: founder effect and premutations. *Proc. Natl. Acad. Sci. USA* 94, 7452-7457. 10.1073/pnas.94.14.7452 9207112 PMC23842

[DMM032706C9] Gérard C., Xiao X., Filali M., Coulombe Z., Arsenault M., Couet J., Li J., Drolet M.-C., Chapdelaine P., Chikh A. et al. (2014). An AAV9 coding for frataxin clearly improved the symptoms and prolonged the life of Friedreich ataxia mouse models. *Mol. Ther. Methods Clin. Dev.* 1, 14044 10.1038/mtm.2014.44 26015982 PMC4362356

[DMM032706C10] Hacein-Bey-Abina S., Garrigue A., Wang G. P., Soulier J., Lim A., Morillon E., Clappier E., Caccavelli L., Delabesse E., Beldjord K. et al. (2008). Insertional oncogenesis in 4 patients after retrovirus-mediated gene therapy of SCID-X1. *J. Clin. Invest.* 118, 3132-3142. 10.1172/JCI35700 18688285 PMC2496963

[DMM032706C11] Hacein-Bey-Abina S., Hauer J., Lim A., Picard C., Wang G. P., Berry C. C., Martinache C., Rieux-Laucat F., Latour S., Belohradsky B. H. et al. (2010). Efficacy of gene therapy for X-linked severe combined immunodeficiency. *N. Engl. J. Med.* 363, 355-364. 10.1056/NEJMoa1000164 20660403 PMC2957288

[DMM032706C12] Herman D., Jenssen K., Burnett R., Soragni E., Perlman S. L. and Gottesfeld J. M. (2006). Histone deacetylase inhibitors reverse gene silencing in Friedreich's ataxia. *Nat. Chem. Biol.* 2, 551-558. 10.1038/nchembio815 16921367

[DMM032706C13] Kakhlon O. and Cabantchik Z. I. (2002). The labile iron pool: characterization, measurement, and participation in cellular processes(1). *Free Radic. Biol. Med.* 33, 1037-1046. 10.1016/S0891-5849(02)01006-7 12374615

[DMM032706C14] Liao Y., Hao Y., Chen H., He Q., Yuan Z. and Cheng J. (2015). Mitochondrial calcium uniporter protein MCU is involved in oxidative stress-induced cell death. *Protein Cell* 6, 434-442. 10.1007/s13238-015-0144-6 25753332 PMC4444813

[DMM032706C15] Llorens J. V., Navarro J. A., Martínez-Sebastián M. J., Baylies M. K., Schneuwly S., Botella J. A. and Molto M. D. (2007). Causative role of oxidative stress in a Drosophila model of Friedreich ataxia. *FASEB J.* 21, 333-344. 10.1096/fj.05-5709com 17167074

[DMM032706C16] Martelli A., Friedman L. S., Reutenauer L., Messaddeq N., Perlman S. L., Lynch D. R., Fedosov K., Schulz J. B., Pandolfo M. and Puccio H. (2012). Clinical data and characterization of the liver conditional mouse model exclude neoplasia as a non-neurological manifestation associated with Friedreich's ataxia. *Dis. Model. Mech.* 5, 860-869. 10.1242/dmm.009829 22736457 PMC3484868

[DMM032706C17] Miranda C. J., Santos M. M., Ohshima K., Tessaro M., Sequeiros J. and Pandolfo M. (2004). Frataxin overexpressing mice. *FEBS Lett.* 572, 281-288. 10.1016/j.febslet.2004.07.022 15304363

[DMM032706C18] Moreno-Cermeño A., Obis E., Bellí G., Cabiscol E., Ros J. and Tamarit J. (2010). Frataxin depletion in yeast triggers up-regulation of iron transport systems before affecting iron-sulfur enzyme activities. *J. Biol. Chem.* 285, 41653-41664. 10.1074/jbc.M110.149443 20956517 PMC3009893

[DMM032706C19] Navarro J. A., Ohmann E., Sanchez D., Botella J. A., Liebisch G., Molto M. D., Ganfornina M. D., Schmitz G. and Schneuwly S. (2010). Altered lipid metabolism in a Drosophila model of Friedreich's ataxia. *Hum. Mol. Genet.* 19, 2828-2840. 10.1093/hmg/ddq183 20460268 PMC7108586

[DMM032706C20] Navarro J. A., Llorens J. V., Soriano S., Botella J. A., Schneuwly S., Martínez-Sebastián M. J. and Moltó M. D. (2011). Overexpression of human and fly frataxins in Drosophila provokes deleterious effects at biochemical, physiological and developmental levels. *PLoS ONE* 6, e21017 10.1371/journal.pone.0021017 21779322 PMC3136927

[DMM032706C21] Ouellet D. L., Cherif K., Rousseau J. and Tremblay J. P. (2017). Deletion of the GAA repeats from the human frataxin gene using the CRISPR-Cas9 system in YG8R-derived cells and mouse models of Friedreich ataxia. *Gene Ther.* 24, 265-274. 10.1038/gt.2016.89 28024081

[DMM032706C22] Pandolfo M. (2009). Friedreich ataxia: the clinical picture. *J. Neurol.* 256 Suppl. 1, 3-8. 10.1007/s00415-009-1002-3 19283344

[DMM032706C23] Pandolfo M. and Pastore A. (2009). The pathogenesis of Friedreich ataxia and the structure and function of frataxin. *J. Neurol.* 256 Suppl. 1, 9-17. 10.1007/s00415-009-1003-2 19283345

[DMM032706C24] Pastore A. and Adinolfi S. (2014). Chronochemistry in neurodegeneration. *Front. Mol. Neurosci.* 7, 20 10.3389/fnmol.2014.00020 24744696 PMC3978368

[DMM032706C25] Pastore A. and Puccio H. (2013). Frataxin: a protein in search for a function. *J. Neurochem.* 126 Suppl. 1, 43-52. 10.1111/jnc.12220 23859340

[DMM032706C26] Pastore C., Adinolfi S., Huynen M. A., Rybin V., Martin S., Mayer M., Bukau B. and Pastore A. (2006). YfhJ, a molecular adaptor in iron-sulfur cluster formation or a frataxin-like protein? *Structure* 14, 857-867. 10.1016/j.str.2006.02.010 16698547

[DMM032706C27] Pelizzoni I., Macco R., Morini M. F., Zacchetti D., Grohovaz F. and Codazzi F. (2011). Iron handling in hippocampal neurons: activity-dependent iron entry and mitochondria-mediated neurotoxicity. *Aging Cell* 10, 172-183. 10.1111/j.1474-9726.2010.00652.x 21108725

[DMM032706C28] Perdomini M., Belbellaa B., Monassier L., Reutenauer L., Messaddeq N., Cartier N., Crystal R. G., Aubourg P. and Puccio H. (2014). Prevention and reversal of severe mitochondrial cardiomyopathy by gene therapy in a mouse model of Friedreich's ataxia. *Nat. Med.* 20, 542-547. 10.1038/nm.3510 24705334

[DMM032706C29] Poburski D., Boerner J. B., Koenig M., Ristow M. and Thierbach R. (2016). Time-resolved functional analysis of acute impairment of frataxin expression in an inducible cell model of Friedreich ataxia. *Biol. Open* 5, 654-661. 10.1242/bio.017004 27106929 PMC4874353

[DMM032706C30] Prischi F., Konarev P. V., Iannuzzi C., Pastore C., Adinolfi S., Martin S. R., Svergun D. I. and Pastore A. (2010). Structural bases for the interaction of frataxin with the central components of iron-sulphur cluster assembly. *Nat. Commun.* 1, 95 10.1038/ncomms1097 20981023 PMC2982165

[DMM032706C31] Quaroni L. and Zlateva T. (2014). Real-time metabolic analysis of living cancer cells with correlated cellular spectro-microscopy. *Anal. Chem.* 86, 6887-6895. 10.1021/ac501561x 24914618

[DMM032706C32] Quaroni L., Zlateva T. and Normand E. (2011). Detection of weak absorption changes from molecular events in time-resolved FT-IR spectromicroscopy measurements of single functional cells. *Anal. Chem.* 83, 7371-7380. 10.1021/ac201318z 21854018

[DMM032706C33] Quaroni L., Zlateva T., Wehbe K. and Cinque G. (2016). Infrared imaging of small molecules in living cells: from in vitro metabolic analysis to cytopathology. *Faraday Discuss.* 187, 259-271. 10.1039/C5FD00156K 27049435

[DMM032706C34] Rai M., Soragni E., Chou C. J., Barnes G., Jones S., Rusche J. R., Gottesfeld J. M. and Pandolfo M. (2010). Two new pimelic diphenylamide HDAC inhibitors induce sustained frataxin upregulation in cells from Friedreich's ataxia patients and in a mouse model. *PLoS ONE* 5, e8825 10.1371/journal.pone.0008825 20098685 PMC2809102

[DMM032706C35] Ristow M., Pfister M. F., Yee A. J., Schubert M., Michael L., Zhang C.-Y., Ueki K., Michael M. D. II, Lowell B. B. and Kahn C. R. (2000). Frataxin activates mitochondrial energy conversion and oxidative phosphorylation. *Proc. Natl. Acad. Sci. USA* 97, 12239-12243. 10.1073/pnas.220403797 11035806 PMC17325

[DMM032706C36] Rötig A., de Lonlay P., Chretien D., Foury F., Koenig M., Sidi D., Munnich A. and Rustin P. (1997). Aconitase and mitochondrial iron-sulphur protein deficiency in Friedreich ataxia. *Nat. Genet.* 17, 215-217. 10.1038/ng1097-215 9326946

[DMM032706C37] Rufini A., Cavallo F., Condò I., Fortuni S., De Martino G., Incani O., Di Venere A., Benini M., Massaro D. S., Arcuri G. et al. (2015). Highly specific ubiquitin-competing molecules effectively promote frataxin accumulation and partially rescue the aconitase defect in Friedreich ataxia cells. *Neurobiol. Dis.* 75, 91-99. 10.1016/j.nbd.2014.12.011 25549872 PMC4358773

[DMM032706C38] Runko A. P., Griswold A. J. and Min K.-T. (2008). Overexpression of frataxin in the mitochondria increases resistance to oxidative stress and extends lifespan in Drosophila. *FEBS Lett.* 582, 715-719. 10.1016/j.febslet.2008.01.046 18258192

[DMM032706C39] Sandi C., Al-Mahdawi S. and Pook M. A. (2013). Epigenetics in Friedreich's ataxia: challenges and opportunities for therapy. *Genet. Res. Int.* 2013, 852080 10.1155/2013/852080 23533785 PMC3590757

[DMM032706C40] Seguin A., Bayot A., Dancis A., Rogowska-Wrzesinska A., Auchère F., Camadro J.-M., Bulteau A. L. and Lesuisse E. (2009). Overexpression of the yeast frataxin homolog (Yfh1): contrasting effects on iron-sulfur cluster assembly, heme synthesis and resistance to oxidative stress. *Mitochondrion* 9, 130-138. 10.1016/j.mito.2009.01.007 19460301

[DMM032706C41] Shoichet S. A., Baumer A. T., Stamenkovic D., Sauer H., Pfeiffer A. F., Kahn C. R., Muller-Wieland D., Richter C. and Ristow M. (2002). Frataxin promotes antioxidant defense in a thiol-dependent manner resulting in diminished malignant transformation in vitro. *Hum. Mol. Genet.* 11, 815-821. 10.1093/hmg/11.7.815 11929854

[DMM032706C42] Vannocci T., Faggianelli N., Zaccagnino S., della Rosa I., Adinolfi S. and Pastore A. (2015). A new cellular model to follow Friedreich's ataxia development in a time-resolved way. *Dis. Model. Mech.* 8, 711-719. 10.1242/dmm.020545 26035392 PMC4486863

[DMM032706C43] Vyas P. M., Tomamichel W. J., Pride P. M., Babbey C. M., Wang Q., Mercier J., Martin E. M. and Payne R. M. (2012). A TAT-frataxin fusion protein increases lifespan and cardiac function in a conditional Friedreich's ataxia mouse model. *Hum. Mol. Genet.* 21, 1230-1247. 10.1093/hmg/ddr554 22113996 PMC3284115

[DMM032706C44] Wang Y., Wang Y., Marcus S. and Busenlehner L. S. (2014). The role of frataxin in fission yeast iron metabolism: implications for Friedreich's ataxia. *Biochim. Biophys. Acta* 1840, 3022-3033. 10.1016/j.bbagen.2014.06.017 24997422

