# Supplementary material for: Adding a temporal dimension to the study of Friedreich's ataxia: the effect of frataxin overexpression in a human cell model
Source: Dis Model Mech. 2018 Jun 25;11(6):dmm032706. doi: 10.1242/dmm.032706 (PMC6031361; doi:10.1242/dmm.032706)
Supplement: Supplementary information [file dmm-11-032706-s1.pdf]

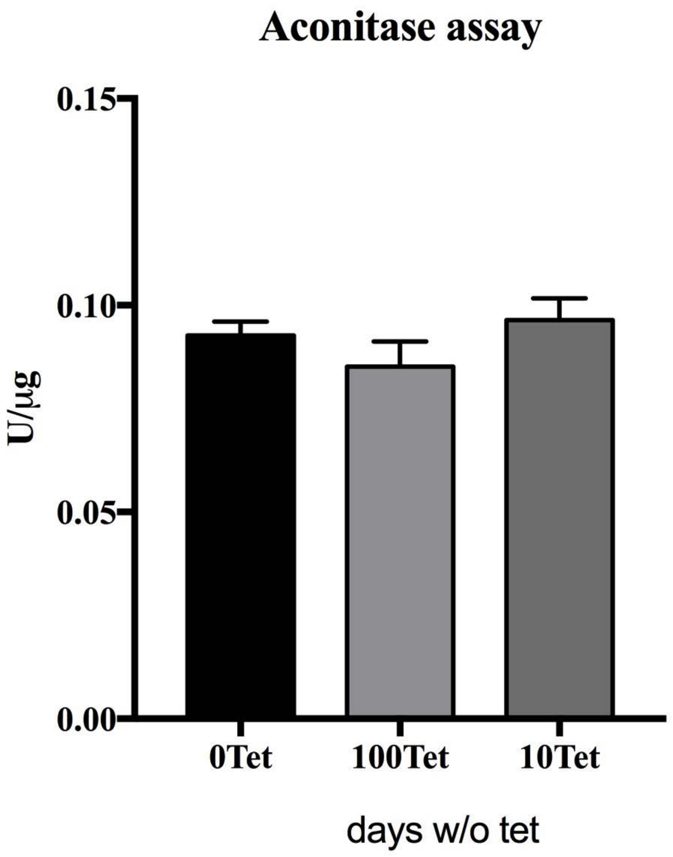

Fig. S1. Tetracycline levels do not affect aconitase activity in wildtype HEK293 cells. Results were pooled from ten technical repeats and are expressed as means (±SD). Data were analysed by ANOVA and Dunnett's post hoc test.

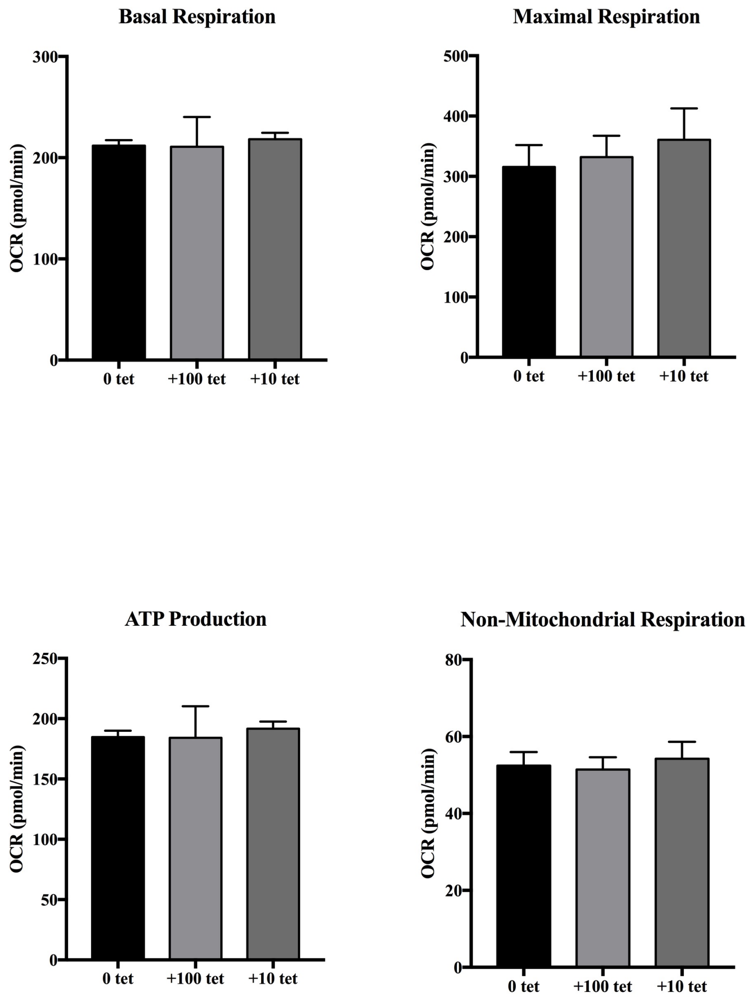

Fig S2. Tetracycline levels do not affect mitochondrial function in wildtype HEK293 cells. The Mitochondrial stress test was performed by measuring OCR variations of cells. Basal respiration, ATP production, maximal respiration and non-mitochondrial respiration were obtained by sequentially treating the samples with oligomycin, FCCP and rotenone/antimycin A compounds. Results are expressed as means (± SD) from ten technical replicates for each condition ($4x10^4$ cells per sample). The recorded signals were normalised against total protein content. Data were analysed by ANOVA and Dunnett's post hoc test.

Disease Models & Mechanisms • Supplementary information

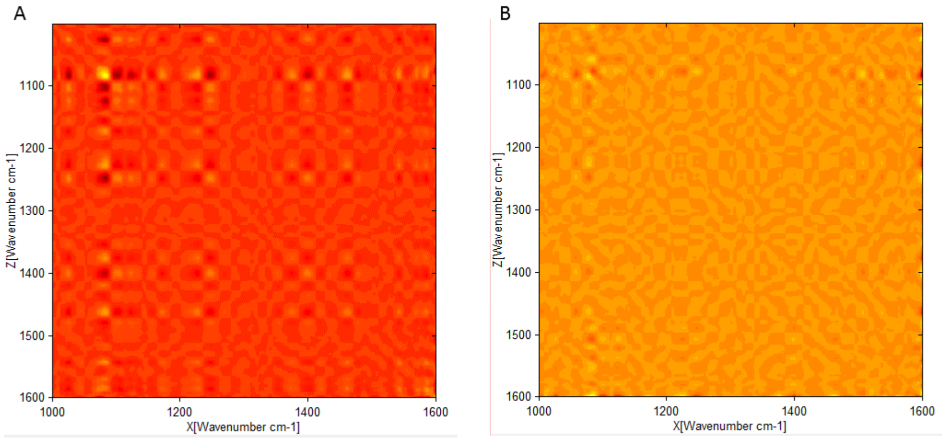

Fig S3. Results of 2D correlation analysis performed on the 2nd derivative of the difference absorbance spectra in Figure 5. (A) Synchronous plot, showing pairwise variations that occur in phase of with opposite phase. (B) Asynchronous plot, showing pairwise variations that occur out of phase.

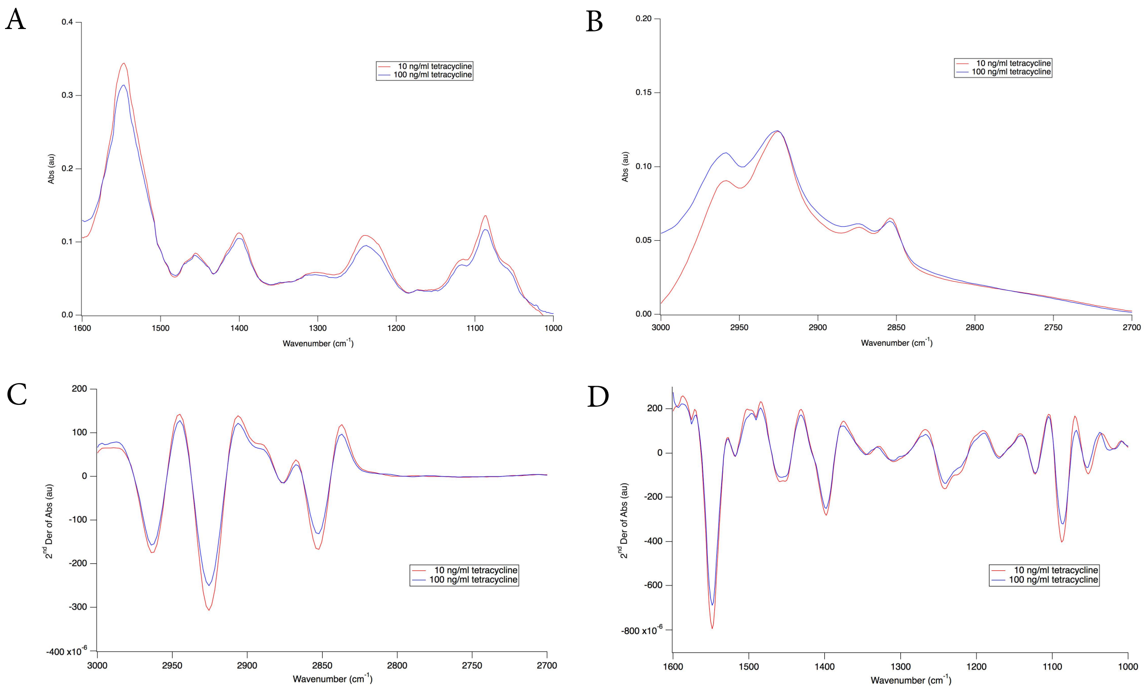

Fig. S4. Comparison of the average spectra of cell cultures grown with different levels of frataxin overexpression. (A,B) Average infrared absorption spectra of the cells grown in the presence of 10 ng/ml (red) and 100 ng/ml (blue) of tetracycline in the range 3000-2700 cm^{-1} (A) and 1600-1000 cm^{-1} (B). (C,D) Second derivatives of the spectra shown in A and B.

Disease Models & Mechanisms • Supplementary information
